# Supplementary material for: Mitochondrial Abnormalities in Induced Pluripotent Stem Cells-Derived Motor Neurons from Patients with Riboflavin Transporter Deficiency
Source: Antioxidants (Basel). 2020 Dec 9;9(12):1252. doi: 10.3390/antiox9121252 (PMC7763948; doi:10.3390/antiox9121252)
Supplement: Supplementary file 1 [file antioxidants-09-01252-s001.pdf]

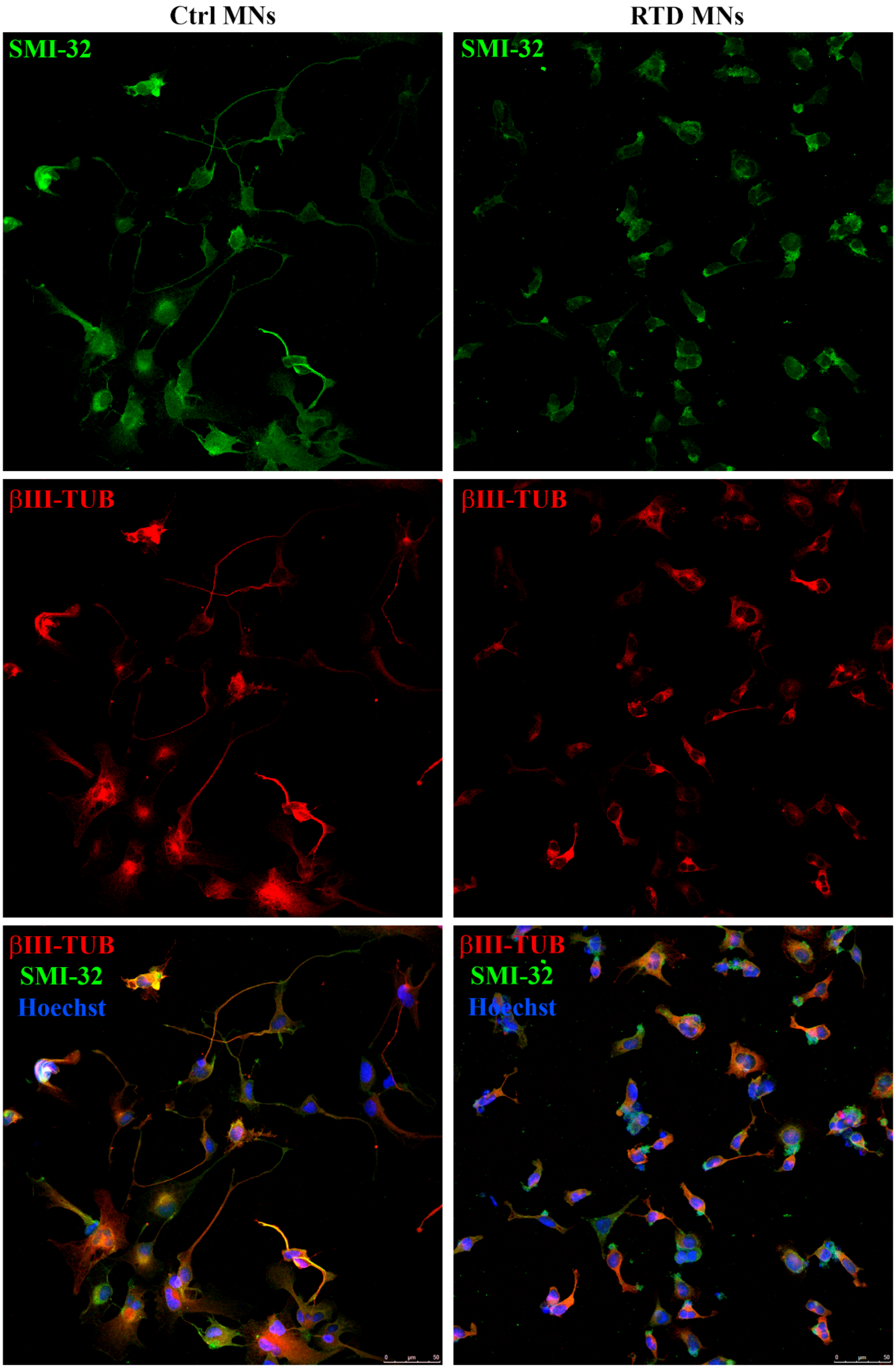

Figure S1. Confocal analysis after immunofluorescence using SMI-32 (green) and βIII-TUB (red) antibodies showing aberrant neuronal morphology of RTD MNs, with respect to Ctrl MNs. Both markers show a patchy distribution in RTD cells, as opposed to the filamentous appearance in healthy neurons. Nuclei are stained with Hoechst (blue).
